# Evolution of joint power across the lifespan during walking

**DOI:** 10.1186/s12984-025-01647-3

**Published:** 2025-06-11

**Authors:** Bernard X. W. Liew, Rachel Senden, David Rugamer, Kenneth Meijer, Qichang Mei, Kim Duffy, Kevin Netto, Matthew Taylor

**Affiliations:** 1 School of Sport, Rehabilitation and Exercise Sciences, University of Essex, Colchester, Essex UK; 2 Department of Physical Therapy, Maastricht University Medical Center, Maastricht, Limburg The Netherlands; 3 Department of Statistics, Ludwig-Maximilians-Universität München, Munich, Germany; 4 Department of Nutrition and Movement Sciences, NUTRIM Research Institute of Nutrition and Translational Research in Metabolism, Maastricht University Medical Centre+, Maastricht, The Netherlands; 5 Faculty of Sports Science, Ningbo University, Ningbo, China; 6 Auckland Bioengineering Institute, The University of Auckland, Auckland, New Zealand; 7 Curtin School of Allied Health, Curtin University, Kent Street, Bentley, WA 6102 Australia

**Keywords:** Biomarkers, Muscles, Lifespan measurement, Motor activity, Biomechanics, Ageing

## Abstract

**Objectives:**

To determine the evolution of lower-limb joint power values during walking across the lifespan.

**Design:**

Series of cross-sectional studies.

**Setting:**

This was a pooled analysis of the individual participant joint power data from six datasets, resulting in a sample size of 629 participants, between the ages of three to 91 years old.

**Main outcome measures:**

Three function-on-scalar regression models were fitted on the outcome measures of joint hip, knee, and ankle power. The covariates of this analysis included sex, age, walking speed, stride length, height, the interaction between age and speed, and a random intercept for different studies.

**Results:**

Ankle push-off (A2) power peaked with a value of 2.46 (95%CI 2.41 to 2.50) W/kg in the 3rd decade of life. Hip early-stance power (H1) peaked in the 1st decade, which followed a sharp decline with age till the 3rd decade. Hip pull-off power (H3) increased sharply to 0.86 (95%CI 0.84 to 0.88) W/kg in the 5th decade and stabilised thereafter with older age.

**Conclusion:**

Ankle push-off power appears to reach maturity in the 3rd decade of life. A strict temporal correspondence between a decline in ankle push-off power (A2) with age and a compensatory increase in hip pull-off power (H3) was not observed, challenging the distal-to-proximal alteration in propulsion strategy commonly attributed to the ageing process.

**Supplementary Information:**

The online version contains supplementary material available at 10.1186/s12984-025-01647-3.

## Introduction

Walking forms a complex, yet integral part of daily life, allowing individuals to play, perform their activities of daily living, work, and navigate within their social environment. Whilst acknowledging the potential for variation, a child first learns to walk between 12 and 14 months. Significant changes occur in the speed and mechanics of walking across the lifespan because of normal maturation, development, and ageing [1–3]. Typical walking speed and mechanics can be negatively affected by many neurological [4], musculoskeletal [5], and cardiovascular [6] disorders, as well as the natural ageing process [2]. Understanding the normative evolution of joint power across the lifespan is particularly crucial, as it can serve as a diagnostic biomarker of impairment and inform the development of therapeutic tools.

**Fig. 1:**
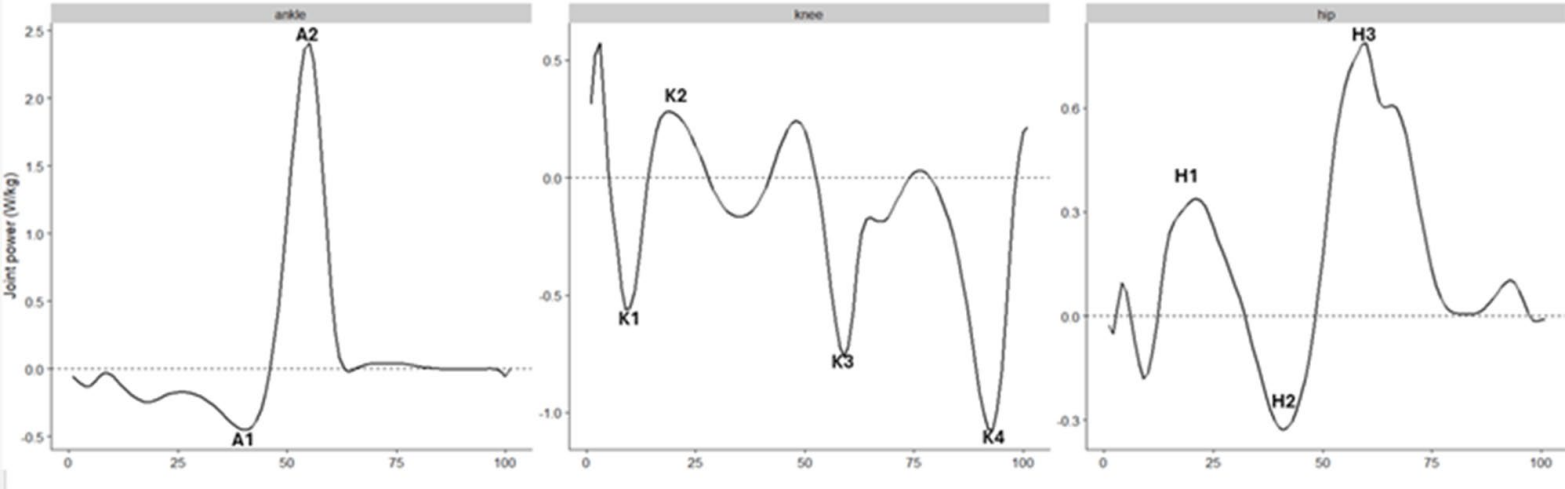
Visualisation of Winter’s discrete joint power indices

**Fig. 2:**
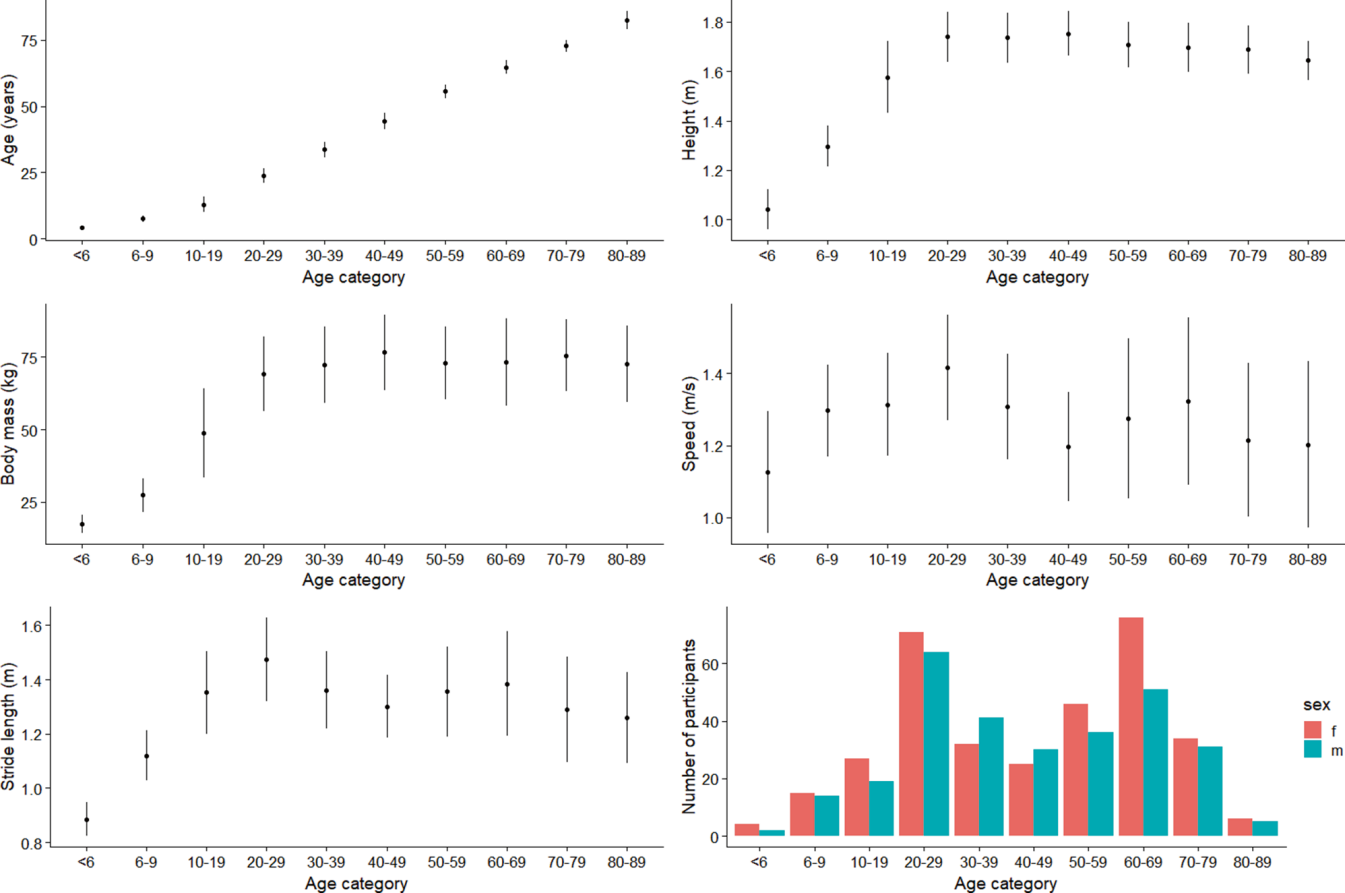
Descriptive characteristics of the included participants. All values represent the mean (one standard deviation as error bars), except the count of the number of participants in each age category

The investigation and quantification of age-typical walking joint power have either focused on the younger adolescent (< 18 years) age groups [1] or on the middle and older adult (≥ 65 years) age groups [7–9], with blended age group studies being rare. Studies of younger age groups aimed to quantify typical developmental patterns in joint power, whilst studies of older age groups aimed to quantify the typical ageing patterns in the joint powers of walking. A description of commonly used discrete joint power indices in the literature can be found in Table 1 and Figs. 1 and 2 [10].

**Table 1:** Description of Winter’s discrete joint power indices

Discrete joint power	Descriptor
A1	A region of negative power, corresponding to eccentric plantar flexor activity at the ankle during midstance and terminal stance
A2	A region of positive power, corresponding to the concentric burst of propulsive plantar flexor activity during pre-swing
K1	A region of negative power, corresponding to eccentric knee extensor activity at during loading response
K2	A region of positive power, corresponding to concentric knee extensor activity during midstance
K3	A region of negative power, corresponding to eccentric activity in the rectus femoris during pre-swing
K4	A region of negative power, corresponding to eccentric activity in the hamstrings during terminal swing
H1	A small region of positive power, not always present, which corresponds to concentric hip extensor activity during loading response
H2	A region of negative power, corresponding to eccentric hip flexor activity during midstance
H3	A region of positive power, corresponding to concentric activity in the hip flexors during pre-swing and initial swing

The maturation of joint power strategies during walking has been thought to show a proximal to distal shift in power generation during walking. An early review by Sutherland observed that the maturation of walking occurred at ~ 4 years [11]. An early study reported that both H1 (hip extensor in early stance) and H3 power generation in early swing, decreased from 4 to 5 years to 18–21 years [12]. The same study reported that A2 ankle push-off power generation increased with age from 4 to 5 years to 18–21 years [12]. A study on 75 healthy children between one and six years old reported that A2 power peaked at 4 years old, H1 power peaked at ~ 2 years old, and H3 power still had not peaked by 6 years old [13].

A previous study analysed joint power during walking in 278 adults between 19 and 86 years old and reported that A2, H1, and H3 powers peaked at 60 years, 20 years, and 70 years, respectively, whilst walking at a common speed of 1 m/s [7]. However, this study did not include children and younger adolescents and had relatively few participants between 30 and 49 years of age [7]. This shortcoming precluded undertaking a lifespan approach towards the analysis of joint power during walking. Another study reported that A2 power did not significantly alter between the 2nd to 6th decades of life, but significantly declined from the 7th decade of life [9]. It is thought that when compared to younger adults, older adults rely more on their hips than their ankles to generate power for walking [14] – also termed a distal-to-proximal shift in motor strategy. However, previous studies reported no evidence of a compensatory effect of hip power for an age-related reduction in joint power [7, 9].

Differences in conclusions regarding the biomechanical maturation of gait have been thought to be due to the presence of confounding factors, such as not controlling for walking speed [15], and the inappropriate scaling of biomechanical data with body anthropometry factors [13, 16]. Some studies have reported that age-associated alterations in joint power distributions were diminished when walking speed and step length were experimentally controlled [15]. Other studies have reported that age-associated alterations become magnified when walking speed is experimentally controlled [17]. Experimentally controlling walking speed may introduce another layer of confounding as it forces an individual to walk with an unnatural walking pattern. Some studies have advocated for normalisation of biomechanical data to anthropometric measures such as height, leg length, and body mass [16, 18–21]. However, other research has expressed concerns over normalising biomechanical data to anthropometric measures [22, 23]. This is because current normalisation procedures could introduce undesirable statistical properties into the biomechanical data [22]. A more appropriate method of considering potential confounding factors when investigating the relationship between age and joint power including them as covariates within regression models [22, 23].

This study aims to undertake, for the first time, a lifespan approach towards the analysis of joint power between the ages of three to 91 years of age. We focus the statistical inference on three primary discrete joint power indices of A2, H1, and H3. The secondary outcomes included the remaining joint power indices of A1, K1 to K4, and H2. Based on the literature, we hypothesised that: (1) A2 power will increase during childhood and peak by the 2nd decade of life, and decline after the 7th decade of life, (2) H1 and H3 powers will decline by the 2nd decade of life and rise again after the 6th decade of life.

## Methods

### Design

We combined individual participant data from six publicly available datasets [7, 24–28] for analysis. Even though methodological variations are present between the included studies, random-effects models are used to account for individual variations in joint powers between studies, allowing to combine data. This approach has also been used in meta-analysis studies (e.g [2]). which routinely pool data from different studies into a random-effects model despite methodological variations in the primary studies (e.g. treadmill [29] and overground [8], the meta-analysis [2]). Similarly, a random-effects model is used to “average” out the between-study variation in joint power.

### Biomechanical protocols overview

The biomechanical protocols used in the six studies can be found in the *supplementary material*, but summarised also in Table 2. Scalar joint power was calculated by the dot product of joint moment and angular velocity. Joint power from each joint and limb was time normalised to 101 cycle points, between two consecutive initial contacts of each limb; and was subsequently normalised to body mass (kg). The average joint power across multiple strides and both limbs was calculated for each participant and speed.

**Table 2:** Brief methodologies of included studies

	Senden	Taylor	Lencioni	Fukuchi	Horst	Schriber
Country	Netherlands	England	Italy	Brazil	Germany	Luxembourg
Inclusion criteria	Typically developing children and healthy adults (3 to 91 yo)	Live independently, be independent walkers, with no surgical procedures (55 to 86 yo)	Healthy, no locomotor disorders (6 to 72 yo)	Healthy, free from lower limb injuries (21 to 84 yo)	Physically active, without gait pathology and free of lower extremity injuries (19 to 67 yo)	Asymptomatic, i.e. healthy and injury free for both lower and upper extremities (19 to 30 yo)
Camera system	12-camera optical motion capture system (Bonita, Vicon, Oxford, UK)	7-camera motion capture system (T20, Vicon, Oxford, UK)	9-camera motion capture system (SMART system, BTS, Garbagnate Milanese, Italy)	12 cameras (Raptor-4, Motion Analysis Corporation, Santa Rosa, CA, USA)	10 cameras (Oqus 310, Qualisys, Gothenburg, Sweden)	10 cameras (Oqus 4, Qualisys, Gothenburg, Sweden)
Camera sampling frequency	100 Hz	100 Hz	60–200 Hz	150 Hz	250 Hz	100 Hz
Force platform system	Instrumented split-belt treadmill (ForceLink, Culemborg)	1 force plate (Type 9281CA, Kistler, Winterthur, Switzerland)	2 force plates (Kistler, Switzerland)	Dual-belt, instrumented treadmill (FIT, Bertec, Columbus, OH, USA)	2 force plates (Type 9287CA, Kistler, Switzerland)	2 force plates (OR6-5, AMTI, Massachusetts, USA)
Force sampling frequency	1000 Hz	1000 Hz	800–960 Hz	300 Hz	1000 Hz	1500 Hz
Footwear	Shod	Shod	Unshod	Unshod	Unshod	Unshod
Surface	Treadmil	Overground	Overground	Treadmill	Overground	Overground
Processing	Markers: LP 2nd Butterworth, ZeroLag, 6 Hz Force: LP 2nd Butterworth, ZeroLag, 6 Hz	Markers: Woltring quintic spline filter (MSE = 10) Force: LP 4th Butterworth, ZeroLag, 10 Hz	Markers: LP 5th Butterworth, ZeroLag, 6 Hz Force: Not reported	Markers: LP 4th Butterworth, ZeroLag, 6 Hz Force: LP 4th Butterworth, ZeroLag, 6 Hz	Markers: LP 4th Butterworth, ZeroLag, 6 Hz Force: LP 4th Butterworth, ZeroLag, 18 Hz	Markers: LP 4th Butterworth, ZeroLag, 6 Hz Force: LP 4th Butterworth, ZeroLag, 18 Hz
Biomechanical model	9-segment human body lower limb, HBM2. Hip 3DOF, knee 1 DOF, ankle 2 DOF	7-segment lower body PiG, each joint 3 DOF	7-segment lower body LAMB, each joint 6 DOF	7-segment lower body, each joint 6 DOF	13-segment full body, each joint 6 DOF	12-segment full body, each joint 6 DOF

### Statistical analysis

All analyses were conducted in R software (v4.3.0) using the *refund* package (v0.1-34) (https://github.com/bernard-liew/Lifespan-Joint-Power). The outcomes represented the joint power trajectories, and the covariates include sex (male or female), age (years), walking speed (m/s), height (m), stride length (m), the study identifier, and the interaction between age and walking speed. Stride frequency was not included as it would introduce a perfectly collinear variable in the model when speed and stride length have already been included. For the knee and hip joints, the joint power is represented as a function of the stride cycle (101 data points, *t*). For the ankle, we represented the joint power as a function of a subset of the stride cycle (data points 25 to 75). This is because ankle power during early stance and mid to late swing was close to zero.

For each of the three joints, we model the expectation of each joint power function yij(t) for every observation *i* and study *j* as a functional additive model.

$$\eqalign{{{\rm{y}}_{ij}}\left( {\rm{t}} \right) &= {{\rm{\beta }}_0}\left( {\rm{t}} \right) + s{\rm{e}}{{\rm{x}}_{{\rm{ij}}}}{{\rm{\beta }}_{{\rm{sex}}}} + {{\rm{f}}_{{\rm{age}}}}\left( {{\rm{ag}}{{\rm{e}}_{{\rm{ij}}}}{\rm{,}}\,{\rm{t}}} \right) \cr&\quad+ {{\rm{f}}_{{\rm{speed}}}}\left( {{\rm{spee}}{{\rm{d}}_{{\rm{ij}}}},\,{\rm{t}}} \right) \cr &\quad+ {{\rm{f}}_{{\rm{strlen}}}}\left( {{\rm{stride\, lengt}}{{\rm{h}}_{{\rm{ij}}}},\,{\rm{t}}} \right) + {{\rm{f}}_{{\rm{ht}}}}\left( {{\rm{heigh}}{{\rm{t}}_{{\rm{ij}}}},\,{\rm{t}}} \right) \cr &\quad+ {{\rm{f}}_{{\rm{age,speed}}}}\left( {{\rm{ag}}{{\rm{e}}_{{\rm{ij}}}},{\rm{ spee}}{{\rm{d}}_{{\rm{ij}}}},\,t} \right) + {{\rm{b}}_{\rm{j}}}\left( {\rm{t}} \right) + {{\rm{\varepsilon }}_{{\rm{ij}}}} \cr}$$

where y_ij_(*t*) is the power value of the respective joint of the *i*th subject and *j*th study at gait point *t*, β_0_(*t*) is the model’s time-varying intercept, β_sex_ a time-independent effect of sex, f(⋅, *t*) indicate functional effects that are estimated to be non-linear both in the direction of the covariate and across time, f_age, speed_(age_ij_, speed_ij_, *t*) is a trivariate smooth effect for the non-linear interaction of age, speed and gait cycle, b_j_(*t*) are time-varying random study intercepts, and ε_ij_ and independent Gaussian error term. The inclusion of a random study intercept enables us to account and adjust for other methodological variations between studies, such as footwear variations.

### Inference

The flexibility of functional regression models presents a limitation in that the reporting of results becomes complex due to the presence of time-varying effects, with nonlinear interactions with the covariates. The recommended approach for reporting would be the predicted mean value of the outcome at given values of each covariate. Here, we reported the predicted mean joint power waveform given the following values of the varying covariates in Table 3, alongside three different walking speeds (0.8, 1.2, 1.4 m/s), and nine different age categories (1st to 9th decade). The values of height and stride length in Table 3 represent the mean values of our participants in this age range. The following walking speeds were used as they represented speeds observed in children (e.g. 0.8 m/s in [1]) and in adults [3]. Lastly, we reported the mean and 95% confidence interval (CI) of the age-associated peak values from A1 to H3. Given the high-dimensionality of the covariates, the inclusion of parametric and non-parametric terms, and the nonlinearity of our models, the present models do not report simple beta-coefficients like in linear regression models. The individual effects of each covariate also become less meaningful to interpret in isolation. However, we still report these smooth and time-varying effects of each covariate, and their 95%CI in the supplementary material.

**Table 3:** Values of the covariates used for model prediction

Age range (years)	Age (years)	Age labels (decade)	Height (m)	Stride length (m)	Sex
< 6	5	1st	1.04	0.88	Male
10–19	15	2nd	1.64	1.34	Male
20–29	25	3rd	1.75	1.47	Male
30–39	35	4th	1.73	1.36	Male
40–49	45	5th	1.74	1.3	Male
50–59	55	6th	1.72	1.35	Male
60–69	65	7th	1.70	1.39	Male
70–79	75	8th	1.69	1.29	Male
80–89	85	9th	1.64	1.26	Male

## Results

**Fig. 3:**
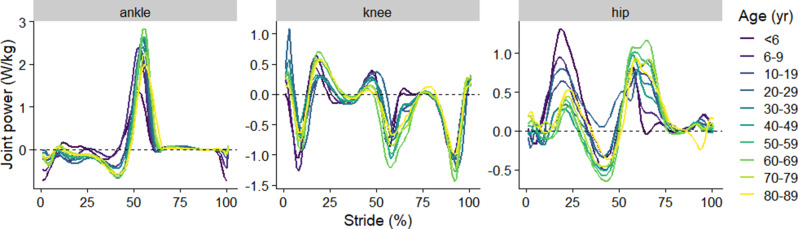
Represents the raw mean joint power trajectories across each age category, whilst, in the supplementary (Figure SM1), the mean joint power trajectories across each age category split by the study are reported

A total of 629 participants from all studies were included in the present analysis. Basic descriptive summaries of the cohort can be found in Fig. 2 (see Table SM1 also in supplementary material). In addition, descriptive summaries of the cohort stratified by study can be found in Figure SM1 (in supplementary material). Figure 3 represents the raw mean joint power trajectories across each age category, whilst in the supplementary material (Figure SM2), the mean joint power trajectories across each age category split by the study are reported.

### Effects of sex on overall joint power

For ankle and hip overall joint powers, male participants were significantly lower than female participants by 0.026 (t = -3.432, *P* < 0.001) and 0.019 (t = -5.095, *P* < 0.001), respectively. For knee overall joint power, male participants were significantly greater than female participants by 0.012 (t = 3.102, *P* = 0.002).

### Primary outcomes of A2, H1, and H3

**Fig. 4:**
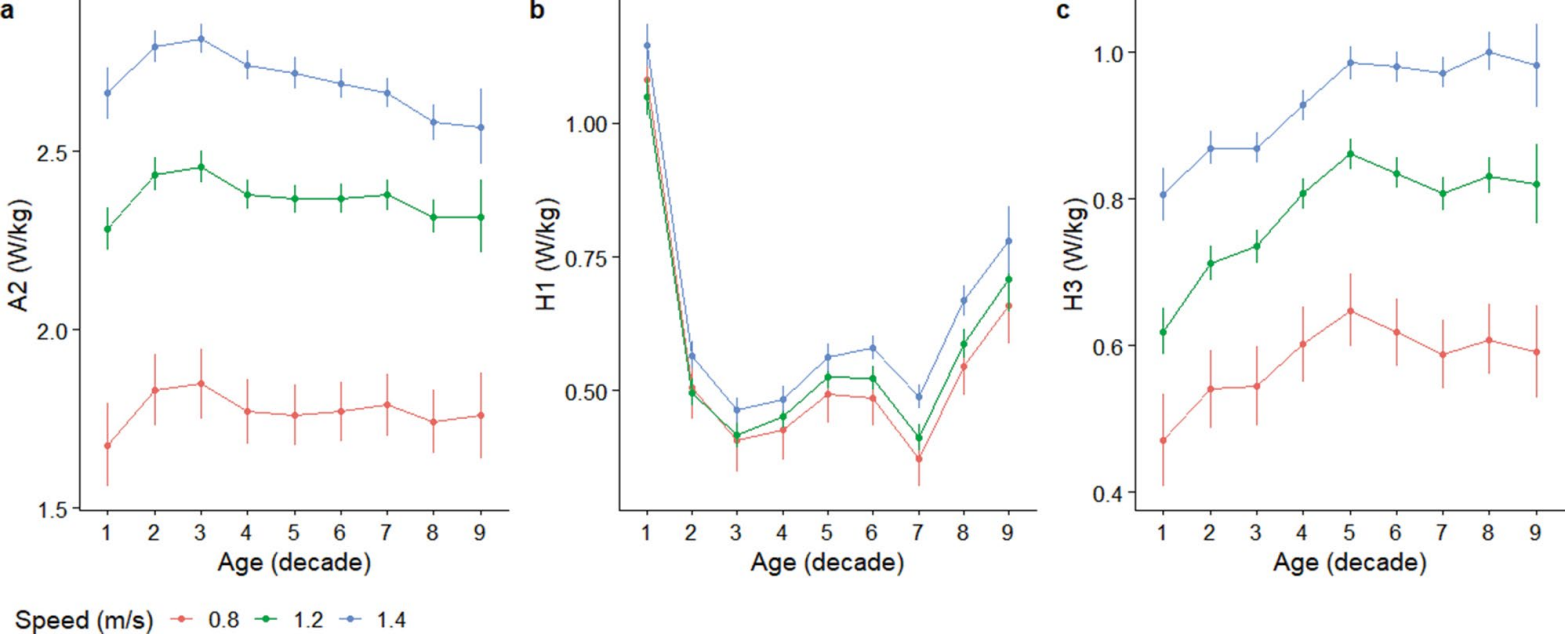
Predicted mean and 95% confidence interval of the three primary joint power outcomes of (a) A2, (b) H1, and (c) H3, using the values of the covariates specified in the manuscript

For a walking speed of 1.2 m/s, A2 peak power was at its lowest of 2.28 (95%CI 2.22 to 2.34) W/kg in the 1st decade and peaked with a value of 2.46 (95%CI 2.41 to 2.50) W/kg in the 3rd decade (Fig. 4a). Thereafter, A2 power declined towards 2.32 (95%CI 2.27 to 2.36) W/kg in the 8th decade (Fig. 4a). These relationships were consistent across the walking speeds predicted. Regardless of walking speed, H1 peaked in the 1st decade, which followed a sharp decline till the 3rd decade (Fig. 4b). At a speed of 1.2 m/s, H1’s lowest value was 0.41 (95%CI 0.39 to 0.44) W/kg in the 7th decade (Fig. 4b). Qualitatively, the trajectory of H1 across the lifespan does not vary at the different speeds that were modelled (Fig. 4b). For H3 at 1.2 m/s, the lowest value was 0.62 (95%CI 0.59 to 0.65) W/kg in the 1st decade. It then increased sharply to a peak of 0.86 (95%CI 0.84 to 0.88) W/kg in the 5th decade and stabilised thereafter with older age (Fig. 4c). The same trend for H3 was observed at different predicted speeds (Fig. 4c).

### Secondary outcomes

**Fig. 5:**
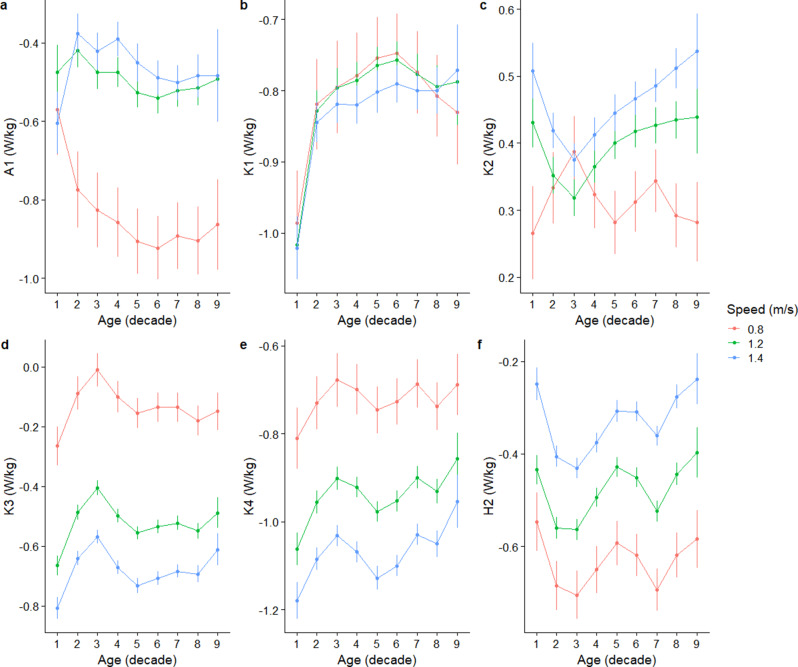
Predicted mean and 95% confidence interval of the secondary joint power outcomes, using the values of the covariates specified in the manuscript

A1 peak power increased sharply from the 1st to the 2nd decade reaching a value of -0.78 W/kg (95%CI -0.87 to – 0.68 W/kg) at a walking speed of 0.8 m/s. Interestingly, at the faster speeds of 1.2 and 1.4 m/s, A1 exhibited a decline in peak power from the 1st to the 2nd decade (Fig. 5a). For K1, the lifespan trajectory demonstrates a very consistent pattern regardless of walking speed (Fig. 5b). The peak K1 power reduced sharply from the 1st to the 2nd decade, and reduced more gradually to the 6th decade; then as age progressed, it stabilised (Fig. 5b). The lifespan trajectories of K2 peak power differed between a slower speed of 0.8 m/s, compared to faster speeds of 1.2 and 1.4 m/s (Fig. 5c). For example, at 1.2 m/s, K2 peak power reduced from the 1st to the 3rd decade, reaching a value of 0.32 (95%CI 0.29 to 0.35) W/kg, before reversing to its greatest value of 0.44 (95%CI 0.39to 0.49) W/kg in the 9th decade (Fig. 5c). In contrast at 0.8 m/s, K2 peak power increased to a maximum of 0.39 (95%CI 0.33 to 0.44) W/kg in the 3rd decade, before declining and fluctuating after the 5th decade (Fig. 5c).

The lifespan trajectories of K3, K4, and H2 retained a similar trajectory across different walking speeds. K3 and K4 peak powers decline in their magnitudes from the 1st to the 3rd decade, before increasing in their magnitudes up to approximately the 5th decade, and then stabilising thereafter (Figs. 5d, e). H2 peak power exhibited multiple fluctuations with age, with a more pronounced increase in magnitude between the 1st to the 2nd decade, and from the 5th to the 7th decade (Fig. 5f).

## Discussion

This work has illustrated the importance of investigating walking as a lifespan continuum. Most ageing walking biomechanics studies tend to compare people only between two age categories, such as between younger vs. older adults [2] and neglect potential alterations between the two age categories. However, the ageing process is heterogeneous, and for adults, this is reflected in the reduction in walking speed from the age of 60 years [30]. Our study shows joint powers change non-linearly with age, and this non-linear alteration persists into older adulthood (i.e. >60 years). This suggests that the homogeneity of walking behavior within older adults should not be assumed.

In partial support of our primary hypotheses, A2 peak power was greatest and H1 power was least in the 3rd decade of life. Contrary to the primary hypotheses, A2 power exhibited a gentle decline after its peak and not after the 7th decade. Also, H1 power exhibited two rises in magnitude – at the 3rd and 7th decades; whilst H3 power had a sharp rise in magnitude after the 1st decade. Beyond the primary hypotheses, an interesting observation was that H1 and K1 power magnitudes across the lifespan did not vary much across the walking speed values used in the prediction. This suggests that both H1 and K1 are less useful as biomechanical biomarkers to detect age-related variations in walking speed.

The age at peak A2 power likely reflects the age of ankle push-off maturation, which is in disagreement with a prior study of 75 healthy children, which reported that ankle dynamics matured around 4 years old [13]. Samson et al. [13] reported that A2 power increased from 1 to 4 years old, and declined from 4 to 6 years old. The present study found that A2 power increased from the 1st to the 3rd decade. The differences in results could be because Samson et al. [13] did not include older individuals, which precluded knowing if a peak in joint power with age represented a transient period or whether maturation was achieved. Our results were congruent with another study, which reported that A2 power increased from < 8 years to 20 years [31].

A2 power decline has been thought to cause the age-related decline in walking speed [14]. Interestingly, when speeds are matched statistically, a sharp decline in A2 power was not observed, but rather a milder decline in A2 power happens from the 3rd to the 4th decade of life. The present findings were consistent with Sloot et al. [9] who reported a Pearson correlation magnitude of only − 0.28 between peak ankle power and age. It appears that healthy older adults can recruit additional ankle power for propulsion to maintain similar walking speeds as younger adults [32].

Older adults have been thought to rely more on their hip muscles for forward propulsion in compensation for a reduction in ankle plantarflexion power, compared to younger adults [14]. Whether an increase in H3 power can be considered an age-related compensatory strategy for an A2 decline may thus be questionable, given that the age at which H3 increases does not coincide with corresponding decreases in A2. One study reported greater H3 in older (78 years) compared to younger adults (26 years) at a walking speed of ~ 1.4 m/s [8], which was supported by the present findings. Contrary to our study, Sloot et al. [9] reported that H3 power declined in older adults (≥ 70 years old) compared to younger adults (30–40 years old). However, walking speed was not controlled for during their analysis [9].

Interestingly, the age-related trajectories of H1 and K1(absorption during loading response) power did not vary qualitatively with walking speed, suggesting that these biomechanical markers do not cause age-related alterations in walking speed. The major hip extensor role is largely to accelerate the centre of mass vertically for body weight support during the period of H1 [33], whilst K1 is for braking and support during the collision phase of walking [33]. Induced acceleration analysis revealed that the gluteus maximus contribution to body weight support increases as a function of walking speed, but the contribution by the gluteus medius remained relatively invariant [33]. Similarly, greater walking speed was associated with greater quadriceps contribution to support and braking during the period of K1 [33]. However, the study by Liu et al. [33] did not control step/stride length variations across different walking speeds. A previous study reported that muscle function during gait is sensitive to stride length variation [34, 35].

Joint power indices like A2 and H3 vary with both walking speed and age, which makes them a challenge to use as biomarkers for ageing. Our results suggest that H1 and K1 may represent speed-invariant gait biomarkers for ageing. These powers occur during early stance and provide a stable platform to propel later during stance. A biomarker is a measurable indicator of health conditions that can inform disease diagnosis, prognosis, treatment, or prevention [36]. If joint powers vary due to age and not walking speed, such as H1 and K1, then it is possible that these could be prognostic biomarkers. This is important since lower-extremity muscle power is a better predictor of functional performance than lower-extremity muscle strength [37]. For example, lower-functioning older adults have significantly reduced H1 power compared to their age-matched, high-functioning counterparts, despite walking speeds being similar [38]. If these power phases are biomarkers, then future work will need to validate these by testing their predictive ability, and then demonstrate the effects of an intervention to ‘improve’ joint power, such as power training (since this increases both force and velocity) [39].

The present study has limitations that should be noted. Even though this study pooled the individual data of 629 participants for analysis, certain age groups are underrepresented in the analysis. Participants aged < 10 years old and > 80 years old are not well represented and would present opportunities for future research and the expansion of our database. A second limitation of the present study is that we did not include data from all public datasets which reported either individual participant data (e.g [40]). or aggregated data (e.g [41]). Incorporation of the present methodology within a formal systematic review framework could represent future research. However, this is especially challenging in biomechanics as not all public datasets provide the required biomechanical data as text results, which makes secondary data pooling quicker. Some public datasets only provide their data in raw motion capture file formats (e.g. C3D) (e.g [42]). This then requires significant resources to prepare the data for analysis. A third limitation of the present study was the cross-sectional design of the included primary studies. Cross-sectional studies cannot distinguish between-subjects relationships (e.g. a greater A2 power on average being associated with greater age on average) from within-subjects relationships (e.g. increasing age by 1 year being associated with a 1 unit change in A2 power).

## Conclusions

Ankle push-off power appears to reach maturity in the 3rd decade of life and exhibits a slow decline in magnitude with age thereafter, which is more apparent at faster walking speeds. A strict temporal correspondence between a decline in ankle push-off power (A2) with age and a compensatory increase in hip pull-off power (H3) was not observed, challenging the distal-to-proximal alteration in propulsion strategy commonly attributed to the ageing process. Interestingly, hip and knee power during the collision period (H1, K1) does not vary significantly with speed but with age. This potentially idealises these peak powers as biomarkers of ageing, without the confounding effects of walking speed that otherwise affect many other candidate biomechanical biomarkers.

## Electronic supplementary material

Below is the link to the electronic supplementary material.

Supplementary Material 1

## Data Availability

No datasets were generated or analysed during the current study.
