# Supplementary material for: Evolution of joint power across the lifespan during walking
Source: J Neuroeng Rehabil. 2025 Jun 11;22:133. doi: 10.1186/s12984-025-01647-3 (PMC12160379; doi:10.1186/s12984-025-01647-3)
Supplement: Supplementary file 1 — Supplementary Material 1 [file 12984_2025_1647_MOESM1_ESM.docx]

Supplementary Material

**Evolution of joint power across the lifespan during walking**

Bernard X.W. Liew^1*^, Rachel Senden^2^, David Rugamer^3^, Kenneth Meijer^4^, Qichang Mei^5,6^, Kim Duffy^1^, Jo Jackson^1^, Matthew Taylor^1^

^1^School of Sport, Rehabilitation and Exercise Sciences, University of Essex, Colchester, Essex, United Kingdom

^2^Department of Physical Therapy, Maastricht University Medical Center, Maastricht, Limburg, The Netherlands.

^3^Department of Statistics, Ludwig-Maximilians-Universität München, Germany

^4^Department of Nutrition and Movement Sciences, NUTRIM Research Institute of Nutrition and Translational Research in Metabolism, Maastricht University Medical Centre+, Maastricht, The Netherlands.

^5^ Faculty of Sports Science, Ningbo University, Ningbo, China

^6^ Auckland Bioengineering Institute, The University of Auckland, Auckland, New Zealand

**Journal category:** Original article

*Corresponding author

E-mail: [bl19622@essex.ac.uk](mailto:bl19622@essex.ac.uk); liew_xwb@hotmail.com. Tel: +44 120 687 3522

**Overview of the studies’ protocols.**

*Senden*

Fifty-five typically developing children [1] and 245 healthy adults [2] spanning the ages of 3 to 91 years old were recruited for this study. Three-dimensional (3D) biomechanical analysis of comfortable speed walking was performed at the Computer Assisted Rehabilitation Environment (CAREN, Motek Medical BV, Amsterdam) system. Participants walked on an instrumented split-belt treadmill (ForceLink, Culemborg, 1000Hz) surrounded by a virtual reality projected on a 180° cylindric screen, while marker trajectories were captured with a 12-camera optical motion capture system (Vicon, Oxford, 100 Hz). The Human Body Lower Limb Model (HBM-II) was used as a biomechanical model. Participants wore standardised gymnastic shoes and a safety harness to prevent falls. [1]. To determine comfortable walking speed for children, the participants had to perform repeated overground walking over a nine-meter walkway, while speed was measured using two movement detection ports. For the adults, the RAMP protocol was used at CAREN where subjects started to walk at 0.5m/s while the speed was gradually increased to 0.01m/s every second until a comfortable speed was reached. This was repeated three times and the average of three repetitions was used as the comfortable speed.

The force plate configurations for the analog data were set at 10 Hz for the low-pass prefilter frequency and 20 N for the force threshold. Marker trajectories and force plate data were filtered with a unidirectional 2^nd^ order Butterworth filter at 6 Hz. Gait event detection was defined based on a combination of heel marker kinematics and force plate data (exceeding the threshold of 50 N) [3]. Custom Matlab scripts were used to check the quality of data and to calculate spatiotemporal parameters, kinematics, and kinetics.

*Study Taylor*

One hundred and forty healthy community-dwelling older adults aged between 55 to 86 years old were recruited for this study [4]. Participants performed overground walking at their comfortable walking speed, over a single in-ground force plate (Kistler 9281CA, Winterthurm, Switzerland). All participants wore their comfortable shoes for testing. A 7-camera VICON T20 motion capture system (Vicon, Oxford, UK, 100 Hz) synchronised to a force plate (1000 Hz) was used. A seven-segment Plug-In Gait (PiG) model was used. Marker trajectories were filtered with a quintic spline filter (Woltring; mean square error of 10) [5], whilst force data were filtered using a low-pass 10 Hz order Butterworth filter. A force plate threshold of 10 N was used to determine gait events. Walking speed was measured via timing gates positioned 2.3 m apart, on either side of the force plate.

*Study Lencioni*

Fifty healthy participants aged between 6 and 72 years old performed overground walking with no shoes at their comfortable speed [6]. Data were collected using two in-ground force plates (Kistler, Switzerland) and a 9-camera motion capture system (SMART system, BTS, Garbagnate Milanese, Italy). Markers trajectories were recorded with a sampling frequency of 60 Hz or 200 Hz, and force plate data at 800 Hz or 960 Hz. A 13-segment model was created for kinetic analysis.

*Study Fukuchi*

Fourty-two healthy adults aged between 21 to 84 years old performed treadmill walking at comfortable speed with no shoes [7]. Biomechanics analysis was conducted using a force-instrumented treadmill (300 Hz, FIT; Bertec, Columbus, OH, USA), and a 12 optoelectronic camera system (150 Hz, Raptor-4; Motion Analysis Corporation, Santa Rosa, CA, USA) [7]. Marker trajectories and ground reaction force (GRF) were low-pass filtered at 6Hz (4^th^ Order, zero-lag, Butterworth) [8]. A seven-segment lower limb, 6DOF joint model was created for analysis. A force plate threshold of 50 N was used to determine gait events of initial contact and toe-off.

Nine participants were excluded from further analysis, resulting in data from 33 participants included. These nine participants had numerous bilateral foot contacts on the same force plate simultaneously, which precluded reliable identification of clean individual limb data.

*Study Horst*

Fifty-seven participants aged between 19 to 67 years old, performed comfortable-speed overground walking without shoes [9]. Data were collected using two in-ground force plates (1000 Hz, Kistler, Switzerland), and motion was captured with 10 optoelectronic cameras (250 Hz, OQUS 310, Qualisys, Sweden). Walking speed was extracted by the mean anterior velocity of the modelled centre of mass (COM) during the periods when the participant was walking over the force plates. Marker trajectories and GRF data were low-passed filtered (4^th^ Order, zero-lag, Butterworth), at 6 Hz and 18 Hz, respectively. A 13-segment full body, 6DOF joint model was created and used for kinetic analysis. A force plate threshold of 20 N was used to determine gait events.

*Study Schreiber*

Fifty healthy participants aged between 19 to 30 years old, performed comfortable-speed overground walking without shoes [10]. Data were collected using two in-ground force plates (1500 Hz, OR6-5, AMTI, USA) and a 10 optoelectronic camera system (100 Hz, OQUS4, Qualisys, Sweden). Walking speed was extracted by the mean anterior velocity of the modelled COM during the periods when the participant was walking over the force plates. Marker trajectories and GRF data were low-passed filtered (4^th^ Order, zero-lag, Butterworth), at 6 Hz and 18 Hz, respectively. A 12-segment full body, 6DOF joint model was created and used for kinetic analysis. A force plate threshold of 20N was used to determine gait events of initial contact and toe-off. Data from one participant were excluded based on visual inspection of the joint power waveforms to reduce extreme values from skewing the data.

Table SM1. Descriptive characteristics of included participants

| **Age category (years)** | **Age (years)** | **Sex (female/male)** | **Height (m)** | **Mass (kg)** | **Stride length (m)** | **Speed (m/s)** |
| --- | --- | --- | --- | --- | --- | --- |
| <6 | 4.17 (0.75) | 4/2 | 1.04 (0.08) | 17.55 (3.12) | 0.88 (0.06) | 1.13 (0.17) |
| 6-9 | 7.55 (1.15) | 15/14 | 1.3 (0.08) | 27.43 (5.77) | 1.12 (0.09) | 1.3 (0.13) |
| 10-19 | 12.89 (2.84) | 27/19 | 1.58 (0.15) | 48.91 (15.35) | 1.35 (0.15) | 1.31 (0.14) |
| 20-29 | 23.76 (2.65) | 71/64 | 1.74 (0.1) | 69.16 (12.89) | 1.47 (0.16) | 1.42 (0.15) |
| 30-29 | 33.6 (2.91) | 32/41 | 1.74 (0.1) | 72.35 (13.13) | 1.36 (0.14) | 1.31 (0.15) |
| 40-49 | 44.29 (3) | 25/30 | 1.75 (0.09) | 76.57 (13.08) | 1.3 (0.12) | 1.2 (0.15) |
| 50-59 | 55.61 (2.62) | 46/36 | 1.71 (0.09) | 72.98 (12.58) | 1.35 (0.17) | 1.28 (0.22) |
| 60-69 | 64.72 (2.65) | 76/51 | 1.7 (0.1) | 73.15 (15.06) | 1.38 (0.19) | 1.32 (0.23) |
| 70-79 | 72.72 (2.3) | 34/31 | 1.69 (0.1) | 75.53 (12.43) | 1.29 (0.19) | 1.21 (0.21) |
| 80-89 | 82.36 (3.47) | 6/5 | 1.64 (0.08) | 72.59 (13.02) | 1.26 (0.17) | 1.2 (0.23) |


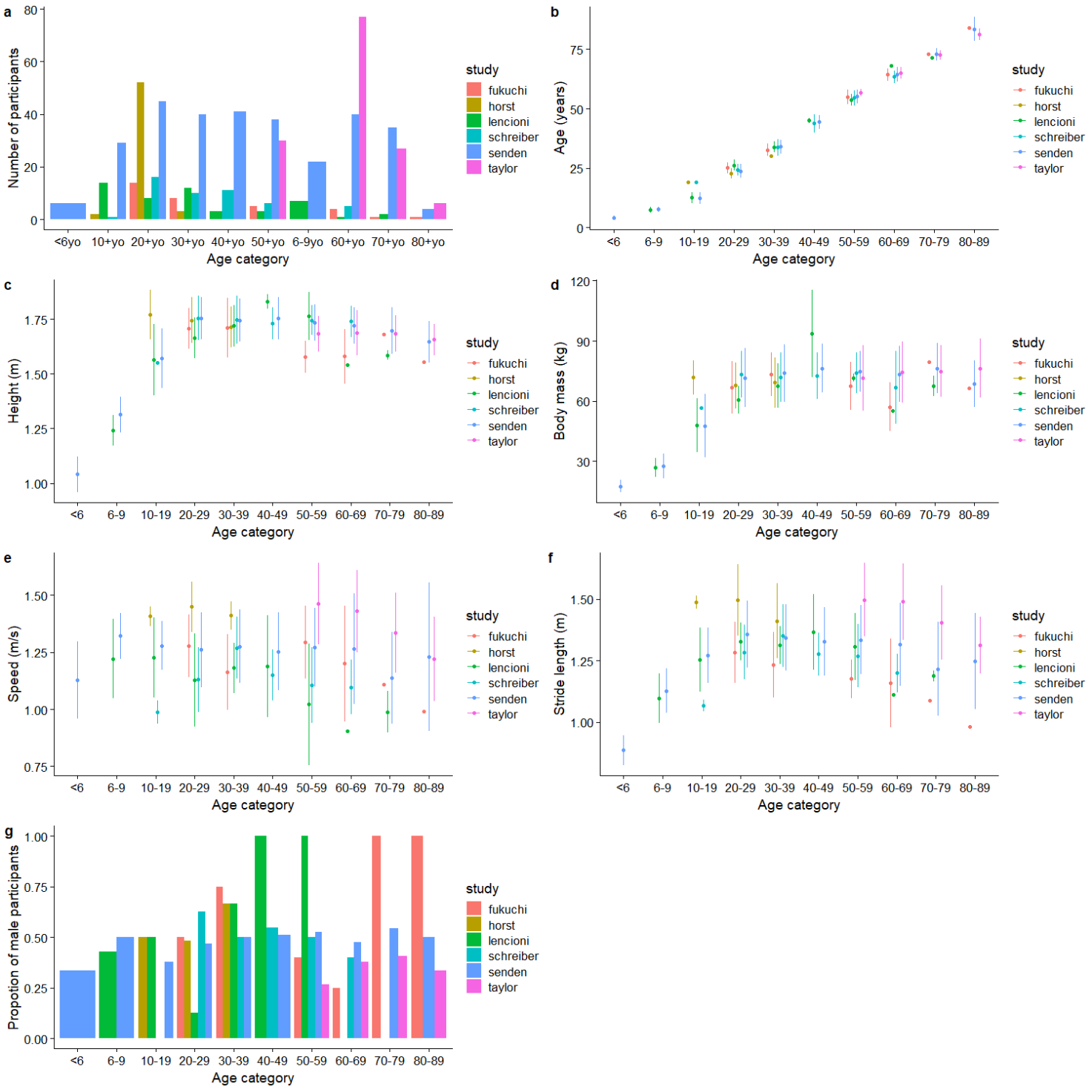


Figure SM 1. Descriptive characteristics of the included participants stratified by study.


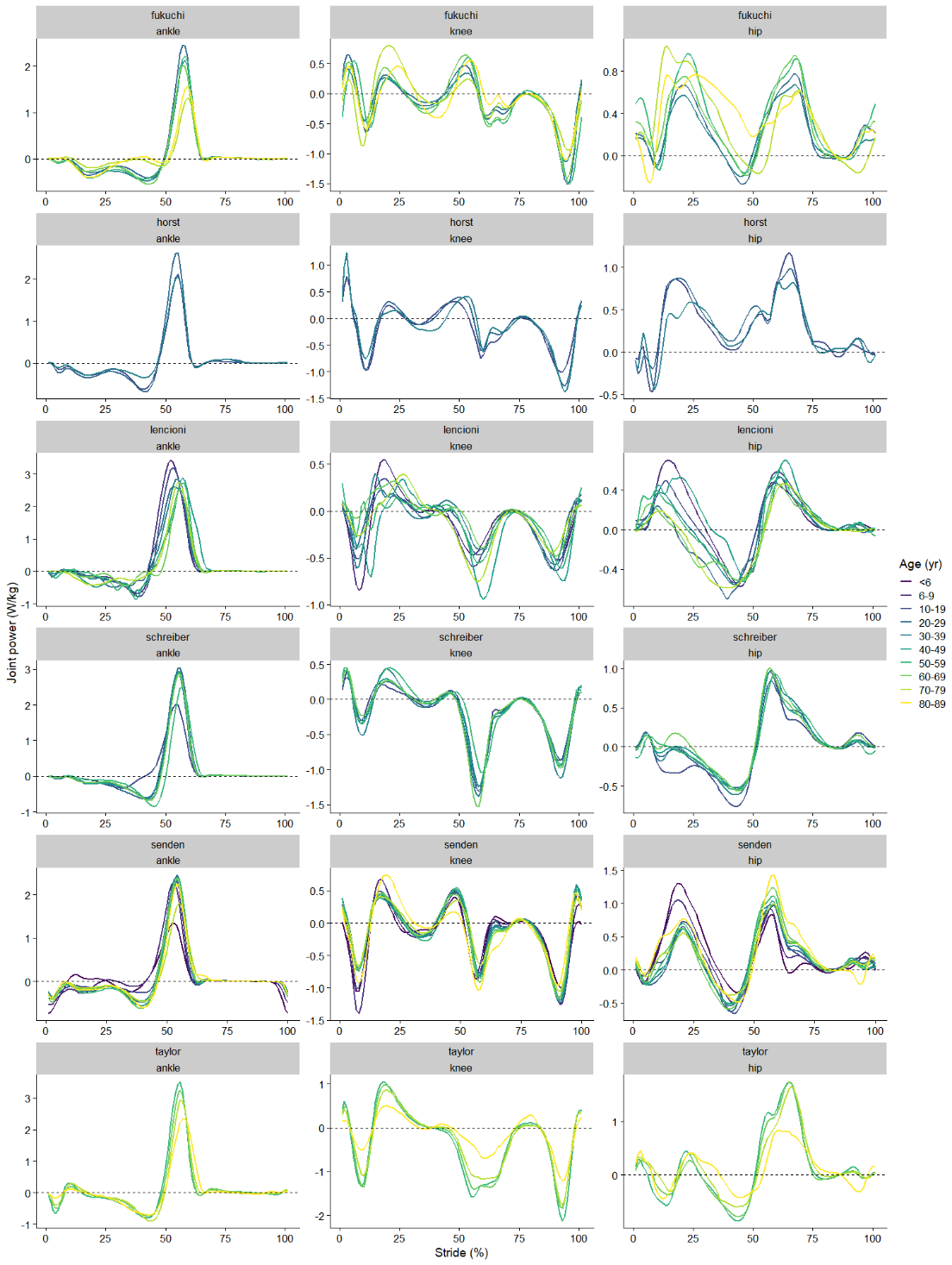


Figure SM2. Joint power across different age categories stratified by studies


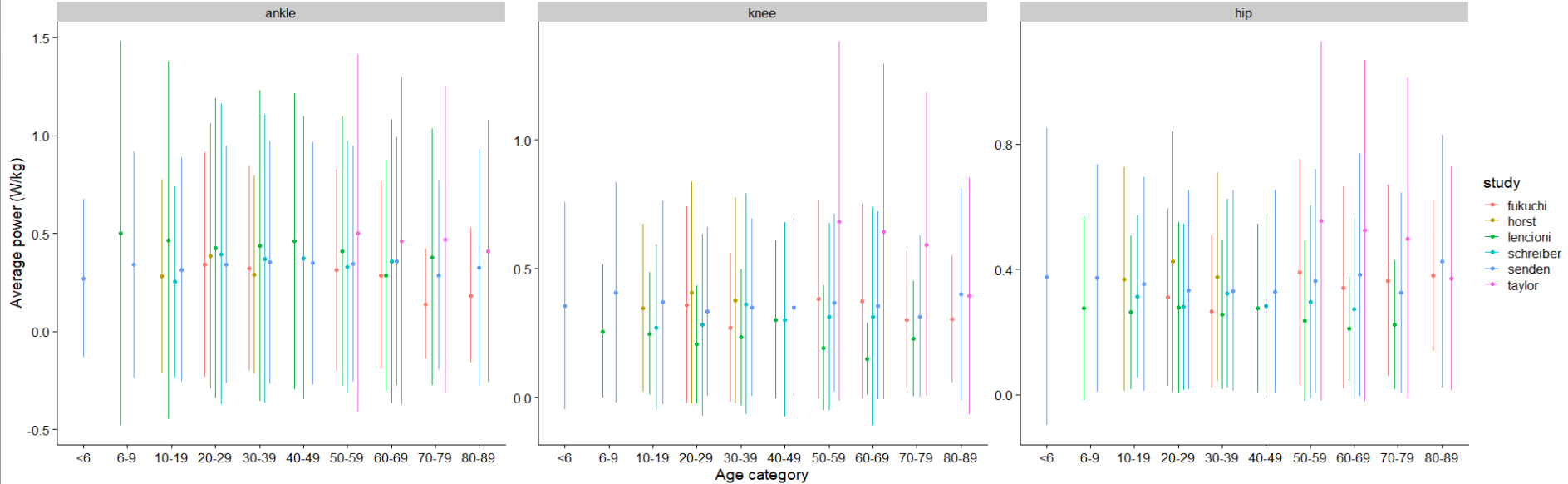


Figure SM3. Mean (error bars as 1 standard deviation) of the average (across the cycle) magnitude of joint power across different age categories stratified by studies.

**Smooth effects and time-varying beta coefficients for ankle power model**

Figure (a) represents the smooth plot between age and cycle, (b) between speed and cycle, and (c) between age and speed on joint power. Figures (d) represent the time-varying beta coefficient of height (m), and (e) stride length (m) on joint power.

**
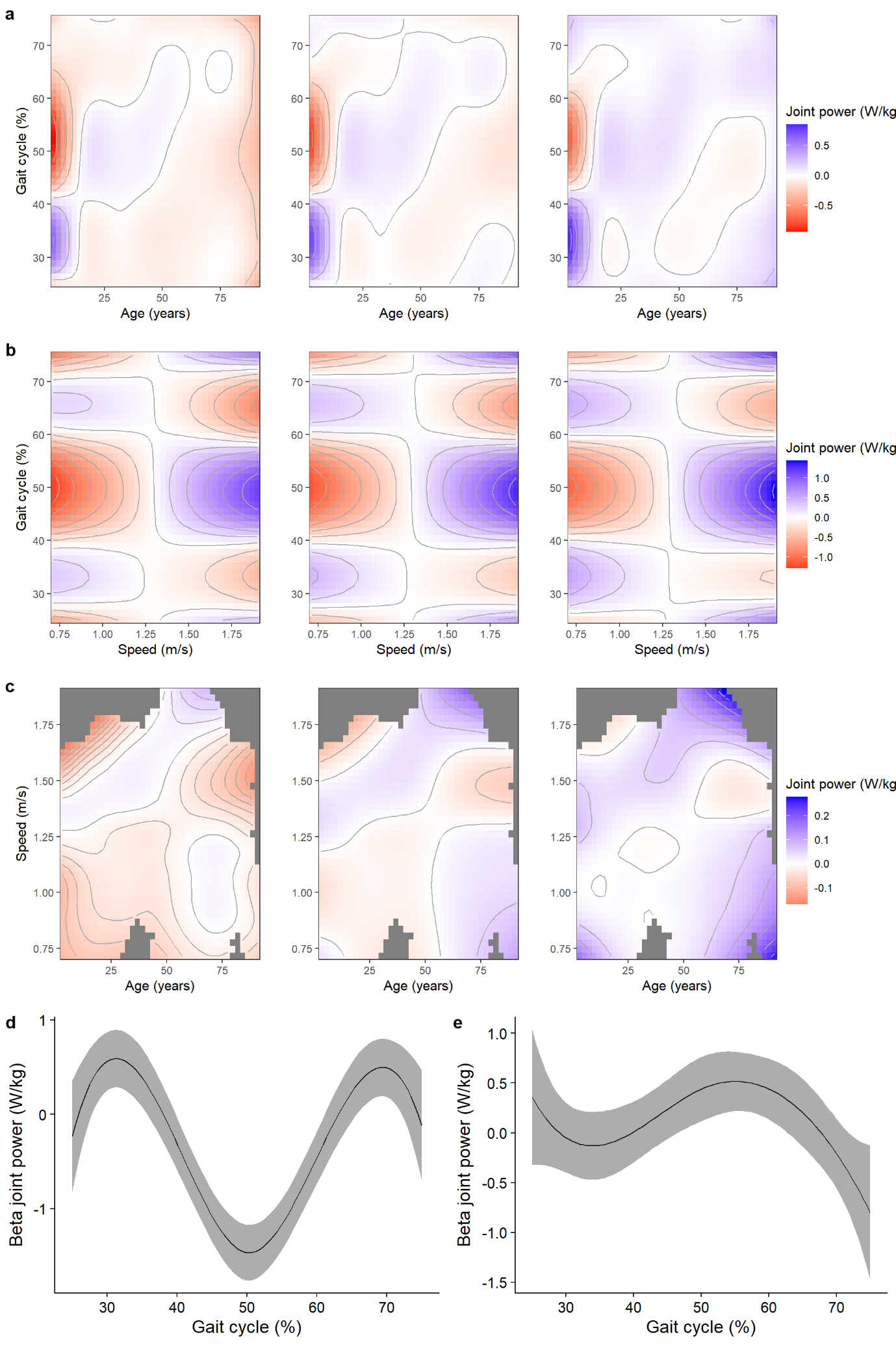
**

**Smooth effects and time-varying beta coefficients for knee power model**

Figure (a) represents the smooth plot between age and cycle, (b) between speed and cycle, and (c) between age and speed on joint power. Figures (d) represent the time-varying beta coefficient of height (m), and (e) stride length (m) on joint power.

**
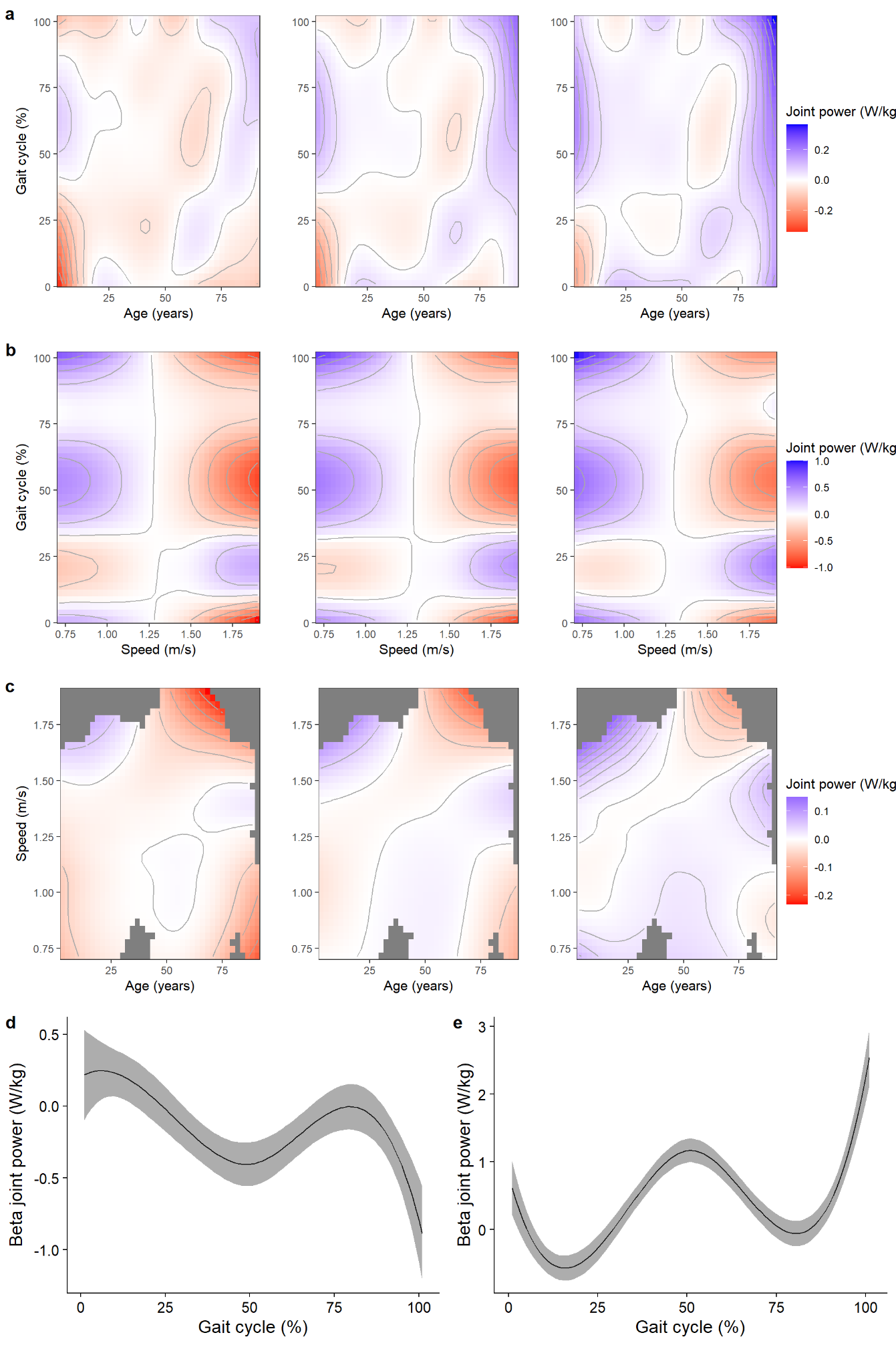
**

**Smooth effects and time-varying beta coefficients for hip power model**

Figure (a) represents the smooth plot between age and cycle, (b) between speed and cycle, and (c) between age and speed on joint power. Figures (d) represent the time-varying beta coefficient of height (m), and (e) stride length (m) on joint power.

**
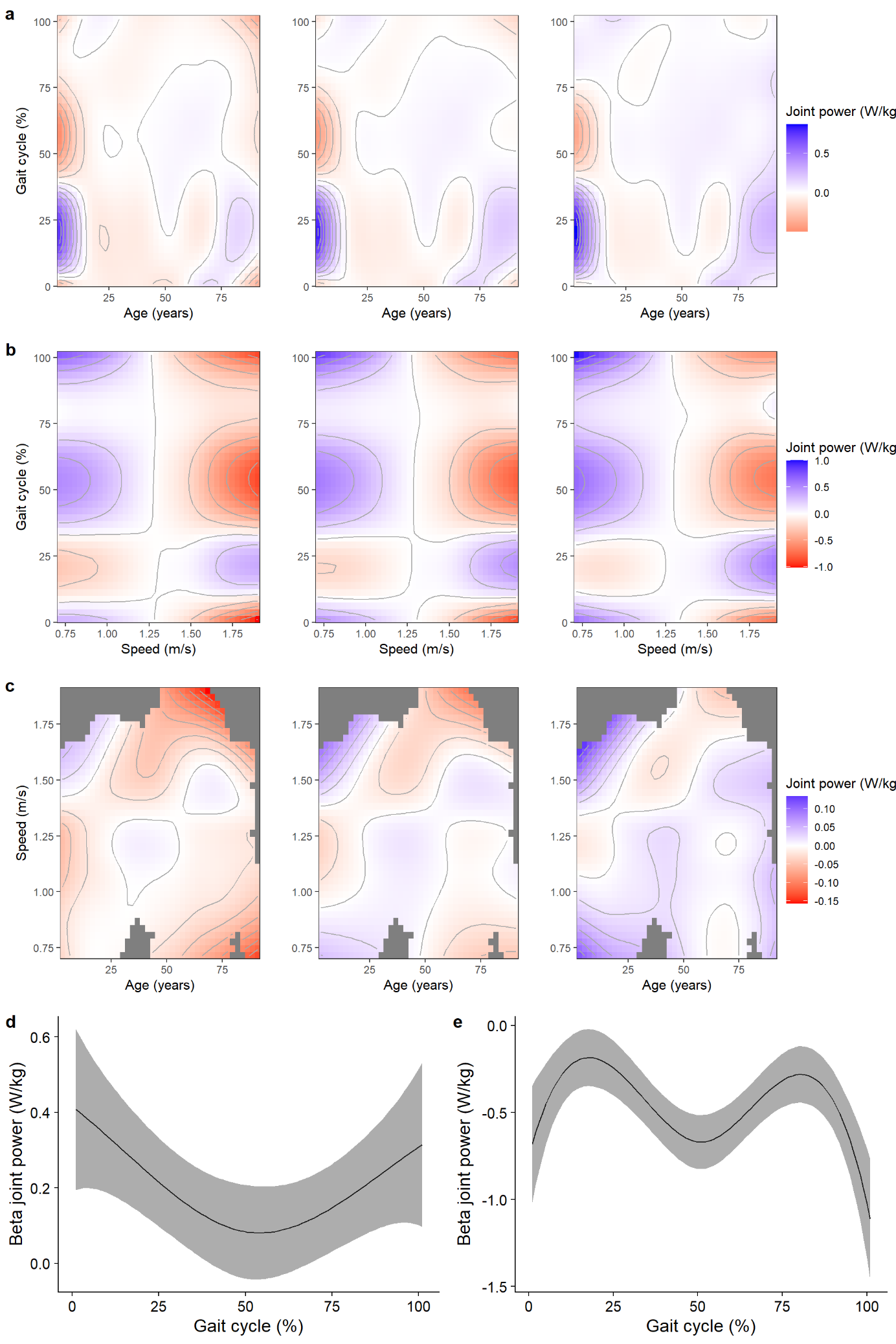
**

**References**

1. Senden R, Marcellis R, Meijer K, Willems P, Lenssen T, Staal H, et al. Dataset of 3D gait analysis in typically developing children walking at three different speeds on an instrumented treadmill in virtual reality. Data Brief. 2023;48:109142.

2. Senden R, Marcellis R, Willems P, Witlox M, Meijer K. Normative 3D gait data of healthy adults walking at three different speeds on an instrumented treadmill in virtual reality. Data Brief. 2024;53:110230.

3. Zeni JA, Jr., Richards JG, Higginson JS. Two simple methods for determining gait events during treadmill and overground walking using kinematic data. Gait Posture. 2008;27:710-714.

4. Liew BXW, Rugamer D, Duffy K, Taylor M, Jackson J. The mechanical energetics of walking across the adult lifespan. PLoS ONE. 2021;16:e0259817.

5. Sinclair J, Taylor PJ, Hobbs SJ. Digital filtering of three-dimensional lower extremity kinematics: an assessment. J Hum Kinet. 2013;39:25-36.

6. Lencioni T, Carpinella I, Rabuffetti M, Marzegan A, Ferrarin M. Human kinematic, kinetic and EMG data during different walking and stair ascending and descending tasks. Sci Data. 2019;6:309.

7. Fukuchi CA, Fukuchi RK, Duarte M. A public dataset of overground and treadmill walking kinematics and kinetics in healthy individuals. PeerJ. 2018;6:e4640.

8. Liew BXW, Morris S, Netto K. Defining gait patterns using Parallel Factor 2 (PARAFAC2): A new analysis of previously published data. J Biomech. 2019;90:133-137.

9. Horst F, Lapuschkin S, Samek W, Müller K-R, Schöllhorn WI. Explaining the unique nature of individual gait patterns with deep learning. Sci Rep. 2019;9:2391.

10. Schreiber C, Moissenet F. A multimodal dataset of human gait at different walking speeds established on injury-free adult participants. Sci Data. 2019;6:111.
